# Association between use of non-aspirin non-steroidal anti-inflammatory drugs and detection of colorectal polyps and cancer in a screening setting

**DOI:** 10.1093/gastro/goag084

**Published:** 2026-10-14

**Authors:** Sara Nafisi, Kristin R Randel, Geir Hoff, Anna L Schult, Edoardo Botteri

**Affiliations:** Department of Research, Cancer Registry of Norway, Norwegian Institute of Public Health, Oslo 0379, Norway; Norwegian Research Centre for Women’s Health, Oslo University Hospital, Oslo 0372, Norway; Department of Colorectal Cancer Screening, Cancer Registry of Norway, Norwegian Institute of Public Health, Oslo 0379, Norway; Department of Colorectal Cancer Screening, Cancer Registry of Norway, Norwegian Institute of Public Health, Oslo 0379, Norway; Department of Research and Development, Telemark Hospital Trust, Skien 3710, Norway; Institute of Clinical Medicine, University of Oslo, Oslo 0372, Norway; Department of Colorectal Cancer Screening, Cancer Registry of Norway, Norwegian Institute of Public Health, Oslo 0379, Norway; Department of Medicine, Vestre Viken Hospital Trust Bærum, Gjettum 1346, Norway; Department of Research, Cancer Registry of Norway, Norwegian Institute of Public Health, Oslo 0379, Norway; Department of Colorectal Cancer Screening, Cancer Registry of Norway, Norwegian Institute of Public Health, Oslo 0379, Norway

**Keywords:** non-steroidal anti-inflammatory drugs, colorectal cancer, colorectal polyps, screening

## Abstract

**Background:**

While substantial evidence supports a protective effect of aspirin against colorectal polyps and cancer, the evidence for non-aspirin non-steroidal anti-inflammatory drugs (NA-NSAIDs) remains limited. We studied the association between the use of NA-NSAIDs and the detection of colorectal polyps and cancer among screening participants.

**Methods:**

We selected participants in the Bowel Cancer Screening trial in Norway—a randomized trial comparing once-only sigmoidoscopy and the biennial faecal immunochemical test (FIT). Using multivariable logistic regression, we estimated odds ratios (ORs) and 95% confidence intervals (CIs) for the association between NA-NSAID use in the 4 years before the screening invitation and detection of colorectal cancer (CRC), adenomas, advanced adenomas, and advanced serrated polyps. Long-term use was defined as ≥2 years of use.

**Results:**

Of 64,889 participants (24,159 sigmoidoscopy, 40,730 FIT), 35,867 (55.3%) were NA-NSAID users, 314 (0.5%) had CRC, 6,208 (9.6%) had adenomas, and 659 (1.0%) had advanced serrated polyps. NA-NSAID users had lower odds of CRC (OR 0.80; 95% CI 0.64–1.00), adenomas (OR 0.94; 95% CI 0.89–1.00), and advanced adenomas (OR 0.87; 95% CI 0.80–0.95) than those with no NA-NSAID prescription. For non-selective NA-NSAIDs, the results were similar to those for any NA-NSAIDs (CRC: OR 0.79, 95% CI 0.63–0.99; adenomas: OR 0.93, 95% CI 0.88–0.99; advanced adenomas: OR 0.87, 95% CI 0.79–0.95). COX2-selective NA-NSAIDs were not associated with CRC or adenomas. Among the long-term NA-NSAIDs users, the ORs were 0.59 (95% CI 0.27–1.26) for CRC, 0.65 (95% CI 0.54–0.79) for adenomas, and 0.59 (95% CI 0.44–0.80) for advanced adenomas. No association was observed between any NA-NSAID use and advanced serrated polyps. The results were consistent across trial arms and sexes.

**Conclusion:**

Our findings suggest that use of NA-NSAIDs, in particular non-selective NA-NSAIDs, might be associated with a reduced risk of adenomas and CRC, but not advanced serrated polyps.

## Background

Colorectal cancer (CRC) is one of the most common cancers worldwide, with >1.9 million new cases reported in 2022 [1]. CRC screening programs are essential for the prevention of CRC [2, 3]. Alongside screening, increasing awareness of modifiable lifestyle risk factors—such as physical inactivity, obesity, alcohol consumption, smoking, and unhealthy diet—is important to reduce the risk of CRC [4]. Over the past two decades, chemoprevention, defined as the use of pharmacological agents to prevent or delay carcinogenesis or suppress cancer progression, has gained increasing attention as a primary prevention strategy [5].

The most widely studied agents in this context are non-steroidal anti-inflammatory drugs (NSAIDs), which inhibit cyclooxygenase (COX) enzymes and thereby reduce the prostaglandin synthesis involved in inflammation and tumorigenesis [6]. NSAIDs are widely used to alleviate pain and inflammation in conditions such as headache, back pain, dental pain, dysmenorrhoea, arthritis, gout, and migraines, as well as to reduce fever. These agents comprise both non-selective NSAIDs, which inhibit COX-1 and COX-2 isoforms, and COX-2–selective inhibitors that preferentially target COX-2 [6]. Aside from the COX-dependent pathway, NSAIDs also inhibit CRC carcinogenesis through COX-independent mechanisms, including the activation of apoptotic mechanisms and suppression of cancer stem cells [7].

Among NSAIDs, aspirin has the most robust evidence for CRC prevention. Regular use has been consistently associated with a reduced risk of CRC, with meta-analyses reporting a 27% reduction in observational studies and a comparable 26% reduction in randomized–controlled trials [8, 9]. In addition to its effects on CRC incidence, observational studies suggest that aspirin use is associated with the primary prevention of colorectal adenomas [10–12].

For non-aspirin NSAIDs (NA-NSAIDs), two meta-analyses of observational studies reported protective associations with CRC, with risk reductions of 26% [13] and 30% [14] among users. Substantial heterogeneity exists across studies, particularly in exposure definitions, which have variably incorporated dose, duration, or frequency of use (e.g. pills per week). The optimal duration of NA-NSAID use for potential protection against colorectal polyps and cancer remains uncertain, ranging from 1 to 5 years across the included studies in those meta-analyses. Additionally, the types of NSAIDs that were studied also differed, from including NA-NSAIDs, to broader categories encompassing aspirin (frequently termed as any NSAIDs), or COX-2 inhibitors exclusively [14, 15]. Furthermore, the potential effect of NA-NSAIDs on the primary prevention of colorectal polyps has rarely been explored.

In this large Norwegian CRC screening trial, we investigated the association between NA-NSAID use and the detection of CRC and colorectal polyps based on histopathological characteristics and by duration of use of NA-NSAIDs. Additionally, we performed stratified analyses for COX2-selective and non-selective NA-NSAIDs to provide more comparable results to those of published studies.

## Methods

### Design and population

This cross-sectional case–control study was conducted by using data from the randomized pilot trial of Bowel Cancer Screening in Norway (BCSN) [12, 16]. In this trial, 140,000 individuals were randomized at a 1:1 ratio and then invited to a once-only sigmoidoscopy or biennial faecal immunochemical test (FIT). We selected 24,159 participants in the sigmoidoscopy arm and 40,930 in the first round of FITs between 2012 and 2017. Of all participants, 3,100 (7.6%) with a positive FIT (>15 micrograms of haemoglobin per gram of faeces) and 2,372 (9.8%) with a positive sigmoidoscopy (any polyp measuring ≥10 mm, at least three adenomas, any advanced adenomas, or CRC) were invited to attend a follow-up colonoscopy. Out of 40,930 in the FIT arm, 200 participants who tested as FIT-positive did not proceed with a colonoscopy and were therefore excluded from the present study.

Participant data from the BCSN trial (age, sex, and screening centre) were linked to prescription records from the Norwegian Prescription Database (NorPD) and socioeconomic data from Statistics Norway through the 11-digit unique Norwegian personal identification number.

This study was reported in accordance with the STROBE guidelines.

### Exposure

NorPD includes information on dispensed medications, such as the Anatomical Therapeutic Chemical (ATC) codes and the number of defined daily doses (DDDs), which is the number of days covered by a prescription, based on the standard daily adult maintenance dose for the primary indication of the drug. Data on exposure to NA-NSAIDs (ATC code M01A) were only available for the 4 years before the invitation. Those with ≥730 cumulative DDDs—corresponding to ≥2 years—were considered long-term users, while those with <730 cumulative DDDs were categorized as short-term users. NA-NSAID use was assessed overall and by COX2-selective versus non-selective agents. Individuals who used both COX2-selective and non-selective NA-NSAIDs were categorized as non-selective NA-NSAID users. Additionally, we performed analyses evaluating the association between the most frequently used individual NA-NSAIDs and the detection of colorectal lesions.

### Screening findings

Screening findings were categorized as follows: no neoplasia (including negative colonoscopies and non-precancerous conditions such as inflammatory bowel disease, haemorrhoids, and diverticula), non-advanced adenomas (adenomas measuring <1 cm, without high-grade dysplasia, and with <25% villous features), advanced adenomas (any adenoma measuring ≥1 cm, with ≥25% villous features, or high-grade dysplasia), non-advanced serrated polyps (hyperplastic polyps measuring <1 cm in diameter and sessile serrated lesions measuring <1 cm without dysplasia), advanced serrated polyps (including traditional serrated adenomas and any serrated polyp measuring ≥1 cm, or with dysplasia) [16], and CRC. Individuals with at least three non-advanced adenomas were classified as having advanced adenoma.

### Statistical analysis

Lesion severity was ordered as CRC > advanced polyps > non-advanced polyps. In rare cases with both advanced adenoma and advanced serrated polyp, classification was subjective. Therefore, we employed distinct multinomial logistic regression models tailored to the lesion of interest. The model included the following outcome categories, arranged by severity: for CRC and adenoma: CRC, adenoma, any serrated polyps, and no lesion; for serrated polyps: CRC, any serrated polyps, adenoma, and no lesion; for non-advanced and advanced adenomas: CRC, advanced adenoma, non-advanced adenoma, any serrated polyps, and no lesion; for non-advanced and advanced serrated polyps: CRC, advanced serrated polyps, non-advanced serrated polyps, adenomas, and no lesion. No prescription of NA-NSAIDs was defined as the reference exposure category and no lesion was defined as the reference outcome category. Odds ratios (ORs) and 95% confidence intervals (CIs) were reported.

All estimates for NA-NSAID use were adjusted for continuous age in years (integer), sex, screening centre, marital status (married or having a cohabitant/not married or not having a cohabitant), immigration status (Norwegian/immigrant), education (primary school/secondary school/≤4 years of university/>4 years of university, missing), employment status (employed/unemployed/outside of workforce or retired), and household income (recategorized in quartiles and missing). Estimates were further adjusted for current use of medications, defined as at least two prescriptions in the 12 months before the screening invitation, including low-dose aspirin, anti-diabetic drugs, antithrombotic agents, cardiac therapy, diuretics, beta-blockers, calcium channel blockers, renin–angiotensin system agents, psycholeptics, antidepressants, and drugs for obstructive airway diseases.

We investigated the heterogeneity of the estimates across the screening arms and sex strata by using the Cochran’s *Q* test. All tests were two-sided, with *P* values of <0.05 being considered statistically significant. Statistical analyses were performed by using R software, version 4.2.3, employing the ‘VGAM’ package for multinomial regression.

## Results

We included 64,889 individuals who attended the screening: 40,730 in the FIT arm and 24,159 in the sigmoidoscopy arm (Table 1). Of all attendees, 35,867 (55.2%) individuals were NA-NSAID users, including 34,093 (52.5%) for non-selective NA-NSAIDs and 1,774 (2.7%) for COX2-selective NA-NSAIDs. In both arms, use of NA-NSAIDs was more common in women than in men and in those with lower education and income than in those with higher education and income. In total, during the screening examinations, CRC was detected in 314 (0.5%) of attendees, adenomas in 3,800 (5.9%), and serrated polyps in 4,170 (6.4%). A total of 2,408 (3.7%) attendees had both adenomas and serrated polyps.

**Table 1:** Attendees’ characteristics and detected lesions by use of NSAIDs in the sigmoidoscopy and FIT arms.

Characteristic	FIT arm		Sigmoidoscopy arm	
	No prescription of NSAIDs [*n* (row %)]	Use of NSAIDs [*n* (row %)]	No prescription of NSAIDs [*n* (row %)]	Use of NSAIDs [*n* (row %)]
All	18,186 (44.7)	22,544 (55.3)	10,836 (44.9)	13,323 (55.1)
Sex				
Female	9,074 (41.9)	12,603 (58.1)	5,138 (41.9)	7,112 (58.1)
Male	9,112 (47.8)	9,941 (52.2)	5,698 (47.8)	6,211 (52.2)
Age groups (years)				
50–55	3,033 (42.5)	4,098 (57.5)	1,819 (45.1)	2,214 (54.9)
56–60	3,941 (43.2)	5,191 (56.8)	2,333 (42.6)	3,141 (57.4)
61–65	3,857 (43.7)	4,967 (56.3)	2,295 (43.4)	2,996 (56.6)
66–70	4,201 (45.8)	4,972 (54.2)	2,481 (46.8)	2,817 (53.2)
>70	3,154 (48.7)	3,316 (51.3)	1,908 (47.0)	2,155 (53.0)
Education				
Primary school	2,756 (39.4)	4,240 (60.6)	1,436 (39.0)	2,245 (61.0)
Secondary (high school)	8,039 (42.2)	11,013 (57.8)	4,859 (42.9)	6,465 (57.1)
≤4 years of university	5,027 (48.6)	5,316 (51.4)	3,048 (47.2)	3,411 (52.8)
>4 years of university	2,260 (54.9)	1,854 (45.1)	1,442 (55.7)	1,148 (44.3)
Missing	104 (46.2)	121 (53.8)	51 (48.6)	54 (51.4)
Employment status				
Employed	11,535 (44.9)	14,158 (55.1)	7,143 (45.0)	8,724 (55.0)
Unemployed	87 (45.1)	106 (54.9)	47 (45.2)	57 (54.8)
Outside of workforce/retired	6,564 (44.2)	8,280 (55.8)	3,646 (44.5)	4,542 (55.5)
Household income (Norwegian Krone)				
Q1^a^ (<559,098)	4,546 (43.0)	6,016 (57.0)	2,517 (44.5)	3,142 (55.5)
Q2 (559,098–833,440)	4,418 (43.6)	5,725 (56.4)	2,559 (42.1)	3,519 (57.9)
Q3 (833,441–1,210,060)	4,468 (44.4)	5,606 (55.6)	2,704 (44.0)	3,441 (56.0)
Q4 (>1,210,060)	4,753 (47.8)	5,194 (52.2)	3,054 (48.7)	3,220 (51.3)
Missing	1 (25.0)	3 (75.0)	2 (66.7)	1 (33.3)
Marital status				
Married/having a cohabitant	14,633 (45.1)	17,842 (54.9)	8,785 (45.2)	10,653 (54.8)
Not married/not having a cohabitant	3,553 (43.0)	4,702 (57.0)	2,051 (43.4)	2,670 (56.6)
Immigration status				
Norwegian	16,949 (44.7)	20,992 (55.3)	10,211 (44.8)	12,599 (55.2)
Immigrant	1,237 (44.4)	1,552 (55.6)	625 (46.3)	724 (53.7)
The most severe finding at the screening				
No lesion	17,165 (44.6)	21,358 (55.4)	7,021 (44.8)	8,653 (55.2)
Serrated polyps	93 (37.8)	153 (62.2)	1,683 (42.9)	2,241 (57.1)
Adenomas	539 (47.9)	587 (52.1)	1,233 (46.1)	1,441 (53.9)
Adenomas and serrated polyps	307 (46.3)	356 (53.7)	820 (47.0)	925 (53.0)
CRC	82 (47.7)	90 (52.3)	79 (55.6)	63 (44.4)

a Q1–Q4: quartiles for household income.

The ORs for CRC detection were 0.80 (95% CI 0.64–1.00) for any NA-NSAID, 0.79 (95% CI 0.63–0.99) for non-selective NA-NSAIDs, and 0.96 (95% CI 0.51–1.83) for COX2-selective NA-NSAIDs (Fig. 1). For short-term use the OR was 0.81 (95% CI 0.65–1.02), whereas for long-term use the OR was 0.59 (95% CI 0.27–1.26). No significant heterogeneity was observed by sex (women: OR 0.73, 95% CI 0.51–1.05; men: OR 0.84, 95% CI 0.63–1.12; *P *= 0.55) or by screening arm (sigmoidoscopy: OR 0.65, 95% CI 0.47–0.91; FIT: OR 0.94, 95% CI 0.69–1.27; *P *= 0.11) (Supplementary Figures S1 and S2).

**Figure 1:**
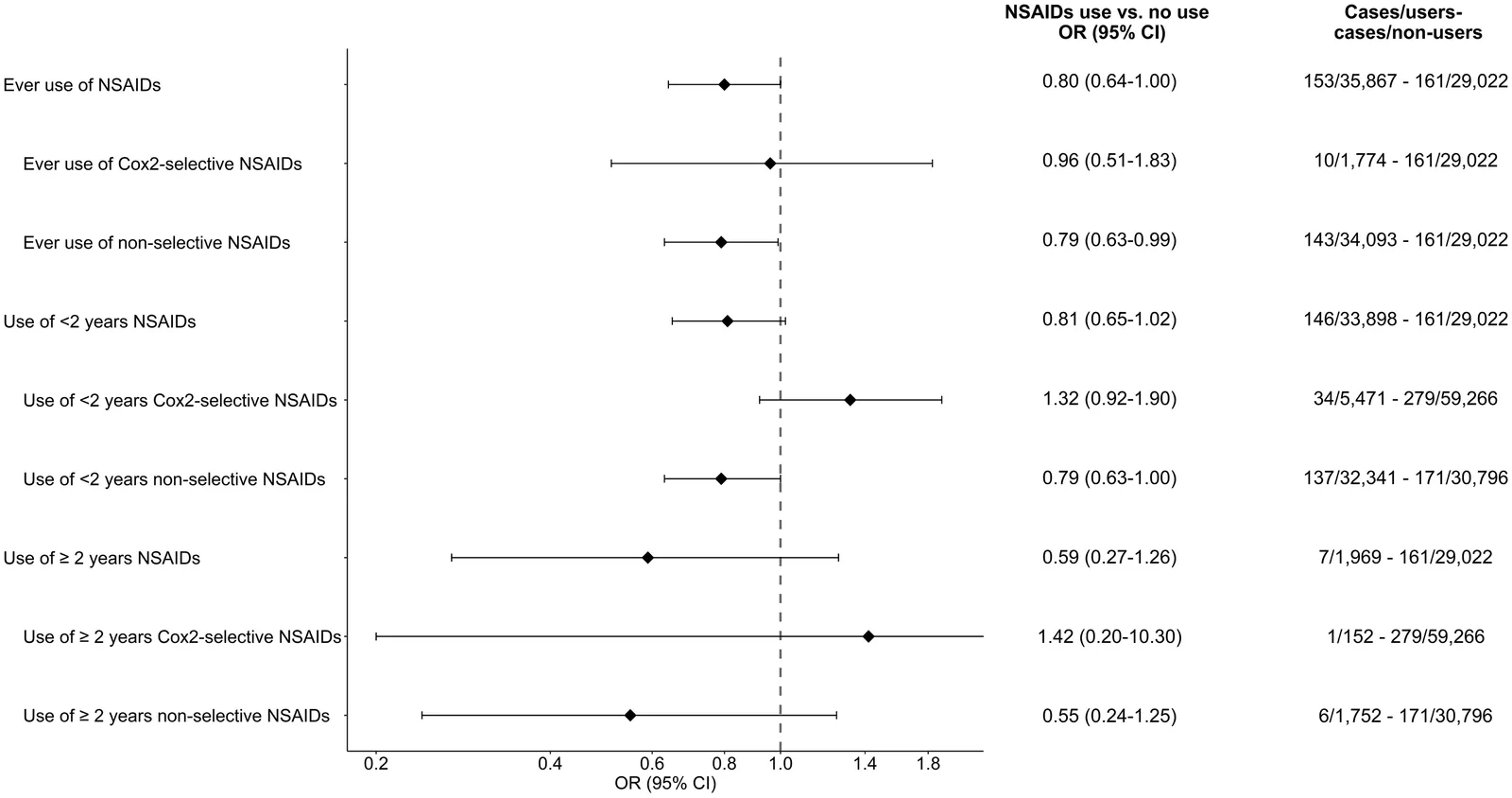
**Association between NSAIDs and the detection of colorectal cancer in screening attendees**. ORs and 95% CIs were adjusted for age, sex, screening centre, marital status, immigration status, education, employment status, household income, and current use of low-dose aspirin, drugs used in diabetes, antithrombotic agents, cardiac therapy, diuretics, antihypertensives including beta-blockers, calcium channel blockers, agents acting on the renin–angiotensin system, psycholeptics, antidepressants, and drugs for obstructive airway diseases. Reference for each estimate is no use of NSAIDs.

For adenoma detection, the ORs were 0.94 (95% CI 0.89–1.00) for any NA-NSAID use, 0.93 (95% CI 0.88–0.99) for non-selective NA-NSAIDs, and 1.10 (95% CI 0.94–1.29) for COX2-selective NA-NSAIDs (Fig. 2). The corresponding estimates for advanced adenomas were 0.87 (95% CI 0.80–0.95), 0.87 (95% CI 0.79–0.95), and 0.90 (95% CI 0.69–1.18). For adenoma detection, we found a strong association between long-term use of any NA-NSAIDs (OR 0.65; 95% CI 0.54–0.79) and non-selective NA-NSAIDs (OR 0.61; 95% CI 0.50–0.74). For advanced adenomas, the ORs were 0.59 (95% CI 0.44–0.80) and 0.54 (95% CI 0.39–0.76). No significant heterogeneity was observed by sex (*P *= 0.94) or screening arm (*P *= 0.50) (Supplementary Figures S3 and S4).

**Figure 2:**
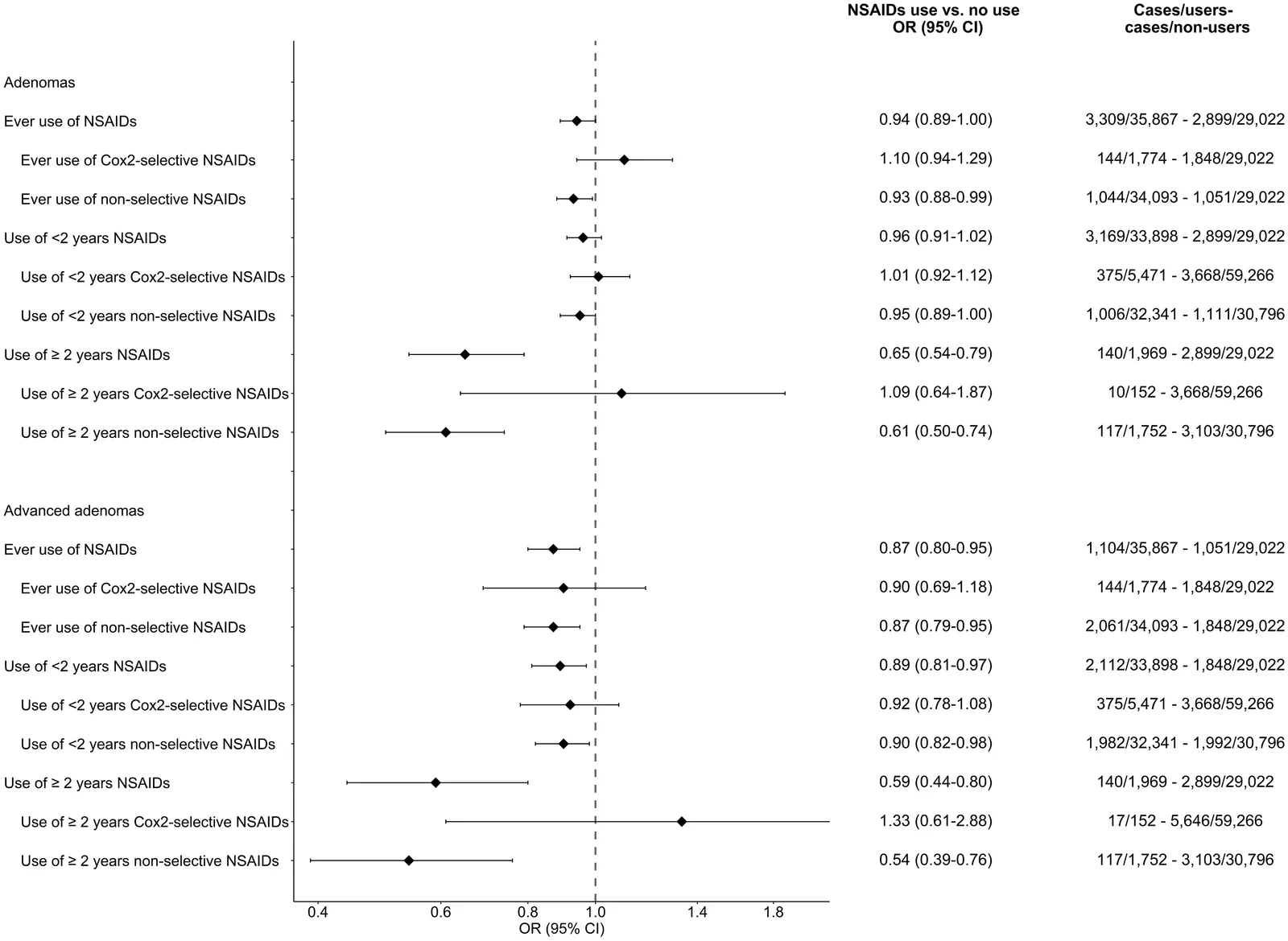
**Association between NSAIDs and the detection of adenomas in screening attendees**. ORs and 95% CIs were adjusted for age, sex, screening centre, marital status, immigration status, education, employment status, household income, and current use of low-dose aspirin, drugs used in diabetes, antithrombotic agents, cardiac therapy, diuretics, antihypertensives including beta-blockers, calcium channel blockers, agents acting on the renin–angiotensin system, psycholeptics, antidepressants, and drugs for obstructive airway diseases. Reference for each estimate is no use of NSAIDs.

Long-term use of NA-NSAIDs, specifically non-selective NA-NSAIDs, was associated with the detection of serrated polyps (Fig. 3). However, we did not observe any association between NA-NSAIDs and the detection of advanced serrated polyps.

**Figure 3:**
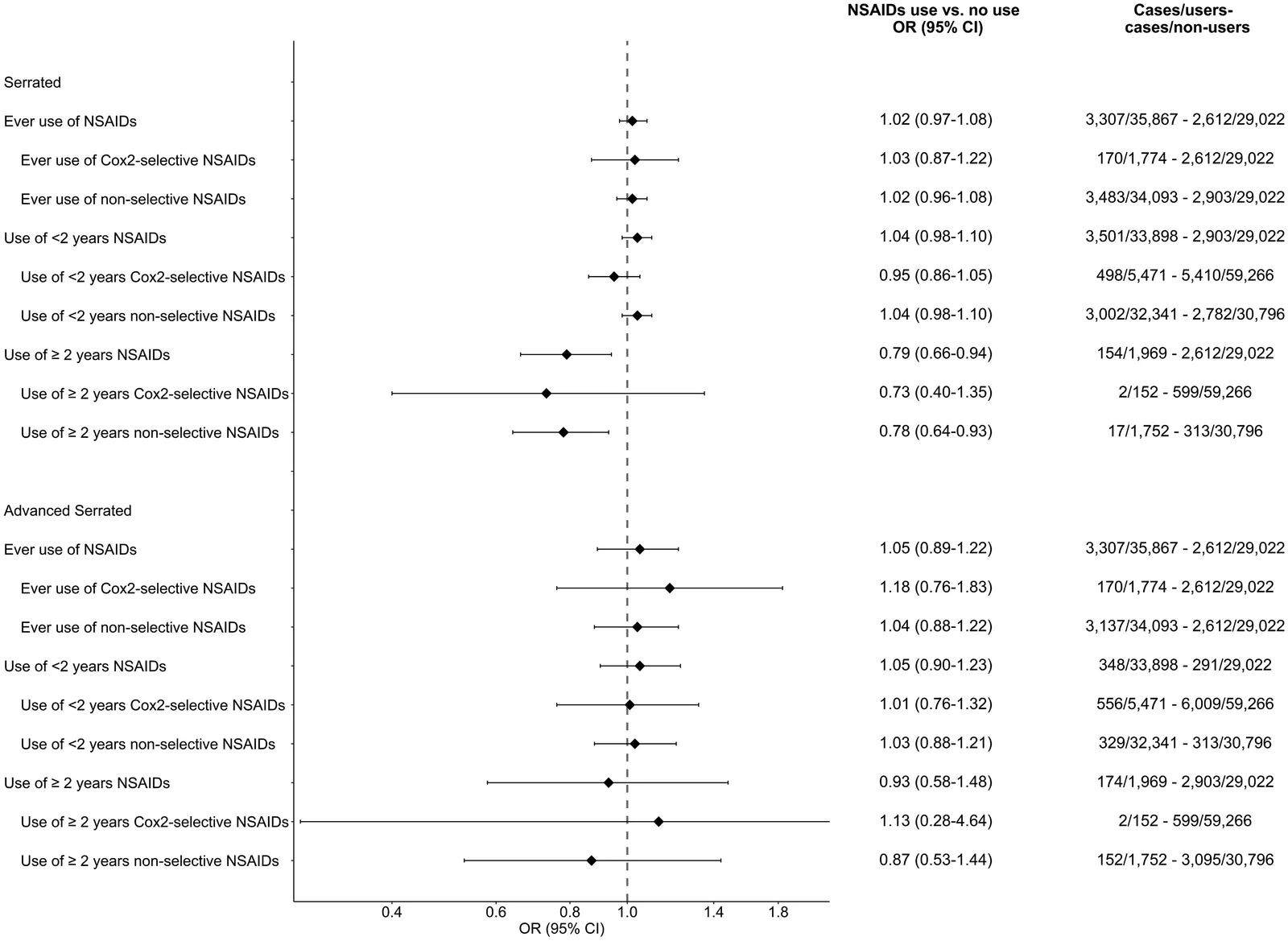
**Association between NSAIDs and the detection of serrated polyps in screening attendees**. ORs and 95% CIs were adjusted for age, sex, screening centre, marital status, immigration status, education, employment status, household income, and current use of low-dose aspirin, drugs used in diabetes, antithrombotic agents, cardiac therapy, diuretics, antihypertensives including beta-blockers, calcium channel blockers, agents acting on the renin–angiotensin system, psycholeptics, antidepressants, and drugs for obstructive airway diseases. Reference for each estimate is no use of NSAIDs.

Among individual NA-NSAIDs, we evaluated diclofenac, ibuprofen, naproxen, etoricoxib, and piroxicam, which were the most frequently used NA-NSAIDs in the study population (62.8%, 31.1%, 17.6%, 13.1%, and 5.4% of screening participants, respectively). Ever use of diclofenac was the only individual NA-NSAID significantly associated with detection of advanced adenomas (OR 0.86, 95% CI 0.78–0.95) (Supplementary Table S1).

## Discussion

In this large screening population, we found evidence suggesting an association between the use of NA-NSAIDs, particularly non-selective NA-NSAIDs, and a lower detection of CRC and adenomas. These associations were more pronounced with longer durations of NA-NSAID use. We did not find any association between the use of COX2-selective NA-NSAIDs and a lower detection of CRC or adenomas. Long-term use of any NA-NSAID and non-selective NA-NSAIDs was associated with a lower detection of serrated polyps, but not advanced serrated polyps.

In this study, we observed a 20% lower detection of CRC among NA-NSAID users. This finding is consistent with the result of a 2019 meta-analysis of cohort and case–control studies, which reported a 26% reduced risk of CRC among NA-NSAID users [13]. Additionally, an older systematic review and meta-analysis commissioned by the US Preventive Services Task Force in 2007 reported a 30% reduced risk of CRC among NA-NSAID users. For adenomas, we observed a 6% lower detection overall and a 13% lower detection of advanced adenomas among NA-NSAID users. Associations were stronger for long-term use (≥2 years), with a 35% lower detection of adenomas and a 41% lower detection of advanced adenomas. These results are in line with those of a 2007 meta-analysis of cohort and case–control studies that reported a 45% reduced incidence of adenoma among individuals who regularly used NA-NSAIDs for ≥1 year [14]. In contrast, our results showed no association between NA-NSAIDs and advanced serrated polyps, consistently with the results of two previous studies, which reported no association between NSAID use and sessile serrated polyps. However, this comparison should be interpreted with caution, as those studies may have included aspirin in the exposure definition [17, 18].

Our findings on NA-NSAID use in relation to adenomas and advanced serrated polyps are consistent with those of preclinical research suggesting that NA-NSAIDs may reduce CRC risk primarily through the inhibition of adenoma development. In 85%–90% of CRC cases, carcinogenesis is initiated by mutations in the adenomatous polyposis coli (APC) gene—a tumour suppressor. These mutations lead to activation of the Wnt/β-catenin signalling pathway, the overexpression of COX enzymes, and uncontrolled cell proliferation into adenomas [19]. NA-NSAIDs exert chemopreventive effects by inhibiting COX enzymes, suppressing inflammation, tumour growth, and immune evasion. Furthermore, NA-NSAIDs can inhibit Wnt signalling through COX-independent mechanisms, including the activation of tumour-suppressive growth factors. By disrupting the Wnt signalling pathway in cancer stem cells, NA-NSAIDs can prevent adenoma formation and progression into cancer [20].

In the stratified analyses by screening arm, we observed no significant difference between the results in the sigmoidoscopy and FIT arms. In 2010, the PLCO trial recruited participants aged 55–74 years to attend sigmoidoscopy screening and found 10% lower adenoma prevalence and 20% lower advanced adenoma prevalence among regular users of ibuprofen, a non-selective NA-NSAID [21]. In our sigmoidoscopy arm, we found a 41% lower detection of adenomas and a 56% lower detection of advanced adenomas among long-term non-selective NA-NSAID users. The stronger association observed in our analysis could be explained by the difference in the exposure definition: we defined long-term use as using ≥730 DDDs of the drug, while, in the PLCO trial, regular use was defined as using at least four pills per month [21].

To the best of our knowledge, this study is among the few studies evaluating the association between NA-NSAIDs and colorectal polyps by histopathological features, providing novel evidence on adenomas and advanced serrated polyps. A key strength is the linkage with population-based registries, ensuring high-quality data on screening outcomes and confounders such as income and education. In addition, linkage with the NorPD minimized bias from self-reported medication use.

The primary limitation of our study was the availability of only 4 years of medication history, which restricted our ability to study longer exposures to NA-NSAIDs. In addition, more detailed stratified analyses according to lesion characteristics, combined with NA-NSAID exposure categories, were limited by small sample sizes and reduced statistical precision. Moreover, the lack of longitudinal follow-up data precluded the assessment of lesion recurrence and CRC prognosis. Another limitation is that NorPD does not capture over-the-counter medication use. Wholesale data from 2017 reported NSAID consumption at 44.9 DDDs/1,000 inhabitants/day, with ibuprofen being the most used (17.2 DDDs/1,000 inhabitants/day), 54% of which was sold over the counter [22]. Over-the-counter sales are likely to introduce exposure misclassification that is unrelated to outcome status (non-differential misclassification), which typically biases estimates toward the null (i.e. ORs closer to 1). Such misclassification may therefore underestimate a potential protective effect by drugs. As over-the-counter purchase generally reflects short-term use, this bias is likely to affect the short-term exposure estimates, whereas long-term use should be less influenced. Another limitation is that we did not have information on lifestyle factors such as diet, physical activity, body mass index, alcohol consumption, and smoking. Considering that unhealthy lifestyle factors are associated with both colorectal lesions and the initiation or prolonged use of NA-NSAIDs [23], this may therefore have resulted in residual confounding and underestimation of the possible protective effect of NA-NSAIDs. To address this limitation, we adjusted the estimates for the use of other medications as proxies for comorbidities associated with lifestyle factors. Moreover, we had no data on the potential side effects of NA-NSAIDs, such as gastrointestinal bleeding and cardiovascular complications, in our population. Despite promising findings, concerns about the cardiovascular and gastrointestinal adverse effects of NA-NSAIDs limit their potential as chemopreventive agents for the general population [7]. Notably, rofecoxib—a COX2-selective NA-NSAID that has been widely studied as a chemopreventive agent against the incidence or recurrence of adenomas [24]—was recalled from the market in 2004 due to increased concerns regarding its cardiovascular adverse effects. Given the adverse effects of NA-NSAIDs, current recommendations for their use as chemopreventive agents are limited to high-risk individuals [25] and most randomised controlled trials on NA-NSAID use for adenoma prevention focus on those groups [26–30]. Our study was conducted among individuals aged 50–74 years who chose to participate in this CRC screening trial. Participation in this trial was influenced by various sociodemographic and health-related factors [31, 32], which might have affected the generalizability of our findings. Furthermore, as only screening participants with a positive FIT or sigmoidoscopy result were referred for a colonoscopy, some lesions might have gone undetected, potentially introducing bias into our estimates. Finally, the results for COX2-selective NSAIDs were not robust, with wide CIs, making it difficult to draw firm conclusions.

In conclusion, this large screening-based study supports the hypothesis that the use of NA-NSAIDs, particularly non-selective agents, may lower the risk of CRC and adenomas, likely through the inhibition of adenoma development, rather than advanced serrated polyps.

## Supplementary Material

goag084_Supplementary_Data

## Data Availability

Due to Norwegian law, we are not allowed to make the data publicly available. Requests to access the data should be directed to the corresponding author.
